# Identification, Validation, and Utilization of Immune Cells in Pancreatic Ductal Adenocarcinoma Based on Marker Genes

**DOI:** 10.3389/fimmu.2021.649061

**Published:** 2021-04-27

**Authors:** Willem de Koning, Diba Latifi, Yunlei Li, Casper H. J. van Eijck, Andrew P. Stubbs, Dana A. M. Mustafa

**Affiliations:** ^1^ Unit of Clinical Bioinformatics, Department of Pathology, Erasmus University Medical Centre, Rotterdam, Netherlands; ^2^ Tumor Immuno-Pathology Laboratory, Department of Pathology, Erasmus University Medical Centre, Rotterdam, Netherlands; ^3^ Department of Surgery, Erasmus University Medical Centre, Rotterdam, Netherlands

**Keywords:** pancreatic ductal adenocarcinoma, marker genes, immune cells, immune microenvironment, mRNA expression

## Abstract

The immune response affects tumor biological behavior and progression. The specific immune characteristics of pancreatic ductal adenocarcinoma (PDAC) can determine the metastatic abilities of cancerous cells and the survival of patients. Therefore, it is important to characterize the specific immune landscape in PDAC tissue samples, and the effect of various types of therapy on that immune composition. Previously, a set of marker genes was identified to assess the immune cell composition in different types of cancer tissue samples. However, gene expression and subtypes of immune cells may vary across different types of cancers. The aim of this study was to provide a method to identify immune cells specifically in PDAC tissue samples. The method is based on defining a specific set of marker genes expressed by various immune cells in PDAC samples. A total of 90 marker genes were selected and tested for immune cell type-specific definition in PDAC; including 43 previously used, and 47 newly selected marker genes. The immune cell-type specificity was checked mathematically by calculating the “pairwise similarity” for all candidate genes using the PDAC RNA-sequenced dataset available at The Cancer Genome Atlas. A set of 55 marker genes that identify 22 different immune cell types for PDAC was created. To validate the method and the set of marker genes, an independent mRNA expression dataset of 24 samples of PDAC patients who received various types of (neo)adjuvant treatments was used. The results showed that by applying our method we were able to identify PDAC specific marker genes to characterize immune cell infiltration in tissue samples. The method we described enabled identifying different subtypes of immune cells that were affected by various types of therapy in PDAC patients. In addition, our method can be easily adapted and applied to identify the specific immune landscape in various types of tissue samples.

## Introduction

Pancreatic cancer is one of the deadliest diseases with a 5-year survival rate of 9% (1). The most prevalent neoplastic disease of the pancreas is pancreatic ductal adenocarcinoma (PDAC) (2). Failure of treatment is partially due to the high heterogeneity of the disease (3). The interaction between cancer and immune cells, known as the immune microenvironment (TME), leads to diverse mechanisms of immune evasion (4). The abundance and composition of tumor-infiltrating lymphocytes (TILs) are fundamental to tumor immunogenicity (5, 6). The variety of TILs and their interaction with pancreatic cancer cells influence tumor progression (7). During the early stages of tumor development, immune cells such as natural killer (NK) and CD8+ T cells facilitate the destruction of immunogenic cancer cells (8). As the tumor evolves, different immune cells infiltrate and have an impact on the tumor’s fate. For instance, high infiltration of CD4+ T cells correlates with a good prognosis (9), while high infiltration levels of regulatory T cells (Tregs) correlate with a poor prognosis (10). In addition, TME and TILs influence the survival of PDAC patients. The high levels of CD8/Tregs ratio correlate with longer survival of the patients (11). Taken together, the accurate determination of the immune infiltration in PDAC tissue samples is important because it provides valuable information regarding how the host immune response interacts with cancer cells. This information can be used in guiding the immunomodulatory approaches to treat PDAC patients.

The gold standard to identify and quantify immune cells in blood samples is Flow cytometry. Immune cells in the blood samples do not need enzymatic disassociation and they can be detected relatively easily after binding to antibodies. However, immune cells in fixed tissue samples, like Fresh-Frozen (FF) or Formalin-Fixed, Paraffin-Embedded (FFPE) samples, are more difficult to quantify by flow cytometry. The methods and enzymes used to dissociate cells in tissue severely harm membranous antigens, makes it more challenging to bind to the antibodies. The preferred method to use for tissue samples is immunohistochemistry (IHC) which showed to be clinically useful (12). However, relatively a lot of tissue sections are needed to measure only a few immune markers. The recent development of this technique enabled multiplexing measurements of various antibodies using one section sample (13). Nevertheless, the number of immune cells that can be identified using IHC-based techniques is still limited and dependents on the availability and accuracy of the antibodies. Alternatively, gene expression profiling is a promising and clinically applicable method for measuring the diversity of TILs in FFPE samples. Various techniques can be used to measure the gene expression profiles of tissue samples. Most of the techniques are based on using enzymatic reactions to synthesize cDNA and amplify it, prior to measure the expression of the genes or sequence the fragments of RNA. However, the targeted gene expression measurements using nCounter^®^ technology (NanoString) enables counting the copies of RNA fragments of tissue samples directly without any enzymatic reactions or amplification steps. It facilitates detecting low abundance targets, down to 0.1–0.5 fM RNA targets, with high sensitivity and high reproducibility [R^2^ > 0.98) (14, 15)]. These features enable determining the immune cell repertoires in FFPE samples. Moreover, many mRNA expression profile databases are available online and can be re-analyzed either to identify the immune cells in a specific cancer type or to validate the findings of a specific analysis. However, an accurate method to identify the TILs based on gene expression per cancer subtype is needed.

To accurately estimate the abundance of the various immune cell populations within the TME, a set of marker genes is needed for each cell type. Previously, a set of immune-specific marker genes were identified to determine cell type across various types of cancer (16, 17). However, gene expression levels are highly affected by the type of tumor. In addition, the marker genes used to identify immune cells may differ in various types of cancer. The aim of this study was to identify a set of marker genes that can be used to characterize the immune landscape in PDAC tissue samples. To that aim, we selected a set of candidate genes (PDAC-cMG), then checked their accuracy to identify immune cells in PDAC samples. Genes that passed our definition criteria were chosen to create the set of marker genes to identify immune cells in PDAC tissue samples (i.e., PDAC-MGICs). To demonstrate the utility of PDAC-MGICs, we applied them to evaluate the effect of therapy on the immune cell infiltration between PDAC patient groups that have been treated with a combination of surgery and neoadjuvant therapy.

## Materials and Methods

### Selecting PDAC Candidate Marker Genes (PDAC-cMG)

A set of candidate marker genes specific for PDAC (PDAC-cMG) were selected based on genes that were previously used to identify immune cells across different types of cancer (n=43) (16). The PDAC-cMG gene list was enriched by genes that were found to identify immune cells in the literature (n=47) (**Table 1** **, Column 2**). A total of 90 candidate genes were included in the PDAC-cMG, represent 23 immune cell types.

**Table 1:** Summary of the candidate gene set and the selected marker genes used to identify immune cell types in PDAC tissue samples.

Column 1	Column 2	Column 3	Column 4
Cell type	Candidate marker genes (PDAC-cMG)	Selected marker genes (PDAC-MGICs)	Default marker genes used in the nSolver^®^ Advanced Analysis
**B cells**	BLK (16), BLNK (16), CCR9 (16), CD19 (16), CD22 (18), CD24 (19), CR2 (16), HLA-DOB (16), HLA-DQA1 (16), MEF2C (16), MS4A1 (16)	BLK, CD19, *CD22*, *CR2*, MS4A1	BLK, CD19, MS4A1, TNFRSF17, FCRL2*, KIAA0125*, PNOC*, SPIB*, TCL1A*
***Plasma B cells***	CD27 (20), CD38 (21), SLAMF7 (22), TNFRSF17 (22)	*CD27*, *CD38*, *SLAMF7*, *TNFRSF17*	
***Regulatory B cells***	CD1D (23), CD5 (23), IL10 (23)	*CD1D*, *CD5*	
**Cytotoxic cells**	GZMA (16), GZMB (16), GZMH (16), KLRB1 (16), KLRD1 (16), KLRK1 (16), PRF1 (16), CTSW (16), GNLY (16)	GZMA, GZMB, GZMH, KLRB1, KLRD1, KLRK1, PRF1	GZMA, GZMB, GZMH, KLRB1, KLRD1, KLRK1, PRF1, CTSW, GNLY, NKG7*
**Dendritic cells**	CCL13 (16), CD1A (24), CD1C (24), CD209 (16), HSD11B1 (16)	*CD1A*, *CD1C*	CCL13, CD209, HSD11B1
***Conventional Dendritic cells 1***	BTLA (24), XCR1 (24), DPP4 (24), THBD (24)	*BTLA*, *XCR1*	
***Conventional Dendritic cells 2***	CD2 (24), ITGAM (24), ITGAX (24)	*ITGAM*, *ITGAX*	
***Plasmacytoid Dendritic cells***	CLEC4C (24), IL3RA (25), NRP1 (24)		
**Macrophages**	CD68 (16), FCGR2A (26)	CD68, *FCGR2A*	CD163, CD68, CD84, MS4A4A*
***Antigen presenting cells***	CCR7 (27), CD80 (28), CD86 (26)	*CD80*, *CD86*	
***M2 Macrophages***	CD163 (29), CD36 (27), MRC1 (27)	*CD163*, *MRC1*	
**Mast cells**	C2 (30), CMA1 (16), CTSG (16), FCER1A (30), MS4A2 (16), PLAU (30), TPSAB1 (16)	MS4A2, TPSAB1	MS4A2, TPSAB1, CPA3*, HDC*, TPSB2*
***Monocytes***	CD14 (27), CD33 (30), TLR2 (31)	*CD14*, *CD33*, *TLR2*	
**Natural Killer cells**	NCR1 (16), XCL2 (16)	NCR1	NCR1, XCL1*, XCL2
**Natural Killer CD56+ dim cells**	IL21R (16), KIR3DL1 (16)	KIR3DL1	IL21R, KIR3DL1, KIR2DL3, KIR3DL2
**Neutrophils**	CSF3R (16), FCGR3A (30), S100A12 (16)	CSF3R, FCGR3A	CEACAM3*, CSF3R, FCAR*, FCGR3B*, FPR1*, S100A12, SIGLEC5*
**T cells**	CD3D (16), CD3E (16), CD3G (16), CD6 (16), SH2D1A (16)	CD3D, CD3E, CD3G, CD6, SH2D1A	CD3D, CD3E, CD3G, CD6, SH2D1A, TRAT1*
***CD4+ T cells***	CD4 (32), SELL (25)	*CD4*, *SELL*	
**CD8+ T cells**	CD8A (16), CD8B (16)	CD8A, CD8B	CD8A, CD8B
**Exhausted CD8+ T cells**	CD244 (16), HAVCR2 (33), LAG3 (16), PDCD1 (33), TIGIT (33)	LAG3, *PDCD1*, *TIGIT*	CD244, EOMES, LAG3, PTGER4
**Helper 1 T cells**	TBX21 (16), ALCAM (25), CD70 (16)	TBX21	TBX21
**Regulatory T cells**	FOXP3 (16), CD274 (34), IDO1 (35), IL2RA (30), TNFRSF18 (30)	FOXP3, *IL2RA*	FOXP3
**CD45+**	PTPRC (16)	PTPRC	PTPRC

The underlined cell types or marker genes are newly defined in comparison to the default of nSolver^®^ Advanced Analysis module of NanoString Technology. Asterisk (*) denotes genes that are not measured by the PanCancer Immune Profiling Panel (Platform GPL19965).

### Downloading Data From The Cancer Genome Atlas (TCGA)

The gene expression profiling data of pancreatic adenocarcinoma (PAAD) from the TCGA database (Level 3 RSEM-normalized, Illumina RNA-seq, Version2) was downloaded (36). The TCGA PAAD dataset is filtered for patients with PDAC primary tumors that received no treatment prior to surgery (n=147). The expression data were log2-transformed prior to pairwise similarity calculations.

### Calculating the Pairwise Similarity

The pairwise similarity statistic between all pairs in the PDAC-cMG per cell type was calculated using an adaptation of Pearson’s correlation metric. The adapted Pearson’s correlation metrics was proven to be better than the simple Pearson correlation (16):

$similarity\left(x,y\right)=\frac{\sum^{}\left(x-\;\bar{x}\right)\left(y-\bar{y}\right)}{\frac{\left(n-1\right)}{2}\left(var\left(x\right)+var\left(y\right)\right)}$

The log2-transformed vectors of the gene expression of two genes are denoted by x and y, where the sample means are denoted by $\bar{x}$ and $\bar{y}$. The sample variance is indicated by var (x) and var (y). This adaptation takes the slope of the correlation into account; hence two ideal cell type marker genes would have a similarity of 1. All calculations were completed using R version 4.0.3.

### Identifying Immune Cells in PDAC Data

The pairwise similarity for all 90 candidate genes was calculated, and genes with high pairwise similarity (≥ 0.6) were selected to be included per cell type in the PDAC-MGICs (**Table 1** **, Column 3**) (16). Each immune cell type was represented by at least two unique genes included in the PDAC-MGICs (37–40). The specificity of the selected gene markers was confirmed by creating heatmaps showing the pairwise similarity of all selected marker genes per immune cell type.

### Validating the Immune Cell Marker Genes Using Published PDAC Profile Data

Previously published data [(41), GEO accession: GSE129492] from 6 PDAC patients who received no systemic therapy prior to surgery (i.e. Surgery Only) were used to validate that PDAC-MGICs are robust and valid immune marker genes in other PDAC cohorts. The database was created by measuring the PanCancer Immune profiles panel. It contained gene expression profiles of 730 immune-related genes and 40 housekeeping genes measured by using nCounter^®^ platform of NanoString technology (Platform GPL19965). The expression level of the 55 genes of the PDAC-MGICs set was checked and confirmed to be higher than the detection threshold in at least 50% of the samples. The gene expression was normalized and log2-transformed using nSolver^®^ (version 4.0) and the Advanced Analysis module (version 2.0) of NanoString technology (NanoString, Seattle, WA, USA). The mean pairwise similarities for the PDAC-MGICs were calculated following the same method that was described earlier.

### Concordance of the New PDAC-MGICs

We validated the marker genes concordance by calculating *p*-values for the cell type gene sets as implemented in the nSolver^®^ Advanced Analysis module (version 2.0). The null hypothesis that a given gene set exhibits no greater cell type-specific-like behavior than a randomly selected gene set of similar size was tested. Therefore, the concordance was calculated for each cell type (i.e., a metric of a gene set’s adherence to the assumption of cell type-specific and consistent expression):

$concordance(X)=\frac{1}{trace(Cov(X))}(p^{-\frac{1}{2}},\cdots .p^{-\frac{1}{2}})Cov(X){\left(p^{-\frac{1}{2}},\ldots p^{-\frac{1}{2}}\right)}^{T}$

The matrix of log2-transformed expression values of the gene set for a specific cell type is denoted by X, and p is the number of genes. The concordance function returns 1 if all genes are perfectly correlated with a slope of 1 and degrade to 0 as this pattern weakens. This concordance is compared to the concordance of 1000 random gene sets of size p, denoted by X0’. The *p*-value equals the proportion of concordance (X0’) values greater than concordance(X0), where concordance(X0) is the concordance of the selected marker genes. The concordance of the gene markers was compared to the default gene markers in nSolver^®^ Advanced Analysis module.

### Validation Using PDAC Samples Affected by Various Types of Neoadjuvant Therapy

The performance of PDAC-MGICs for samples affected by treatments is tested for 18 samples of patients that were subjected to different neoadjuvant therapy prior to surgery: 6 patients received FOLFIRINOX chemotherapy, 6 patients received FOLFIRINOX + stereotactic body radiotherapy (SBRT), and 6 patients received FOLFIRINOX + conventional radiotherapy (XRT) (41). The samples were matched based on lymphovascular invasion and perineural invasion. The database was created by measuring the PanCancer Immune profiles panel. It contained gene expression profiles of 730 immune-related genes and 40 housekeeping genes measured by using nCounter^®^ platform of NanoString technology (GEO accession: GSE129492). Gene expression profiles were normalized and log2-transformed using nSolver^®^ and the Advanced Analysis module, and the pairwise similarity and concordance were calculated as described earlier.

To identify immune cells within the nSolver^®^ advanced analysis module, genes that are annotated to define immune cells within the probe annotation file (provided by NanoString) were changed. After that, the modified probe annotation file was uploaded to a new analysis file, and the average of all genes confirmed to identify a specific immune cell was calculated resulting in a score of a cell type. The scores of cells were compared between the groups of interest, and the significance was calculated using a *t*-test between the groups.

### Utilization of PDAC-MGICs

The clinical utility is demonstrated by uploading our defined marker genes PDAC-GMICs in the nSolver^®^ Advanced Analysis module to identify immune cells in the “cell type profiling” section. The PDAC-GMIC set was used to assess the composition of the immune microenvironment for all patients in GSE129492 (41). The abundance of the immune cell types is represented by a cell score, which is the average log2-transformed expression values of their corresponding marker genes. To correct for the total tumor-infiltrating immune cells per patient, the abundance was calculated relatively to the CD45+ cells. The relative abundance of a cell type in a group of patients is the average log2-scale expression of the marker genes divided by the average log2-scale expression of CD45+. To demonstrate the impact of changing the definition of cells, the relative cell abundances based on the default marker genes of the nSolver^®^ Advanced Analysis module were compared to the relative cell abundance based on PDAC-MGICs.

**Figure 1:**
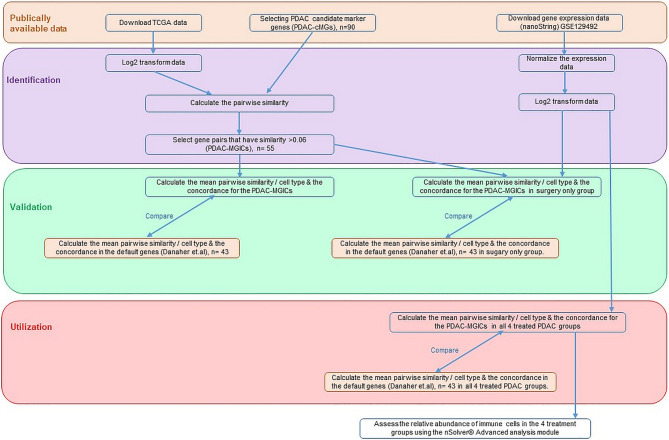
An overview of the method used to determine the definition of immune cells in PDAC tissue samples.

An overview of our method is presented in (**Figure 1**).

## Results

### PDAC-MGICs Enabled Identifying 22 Immune Cell Types

**Figure 2:**
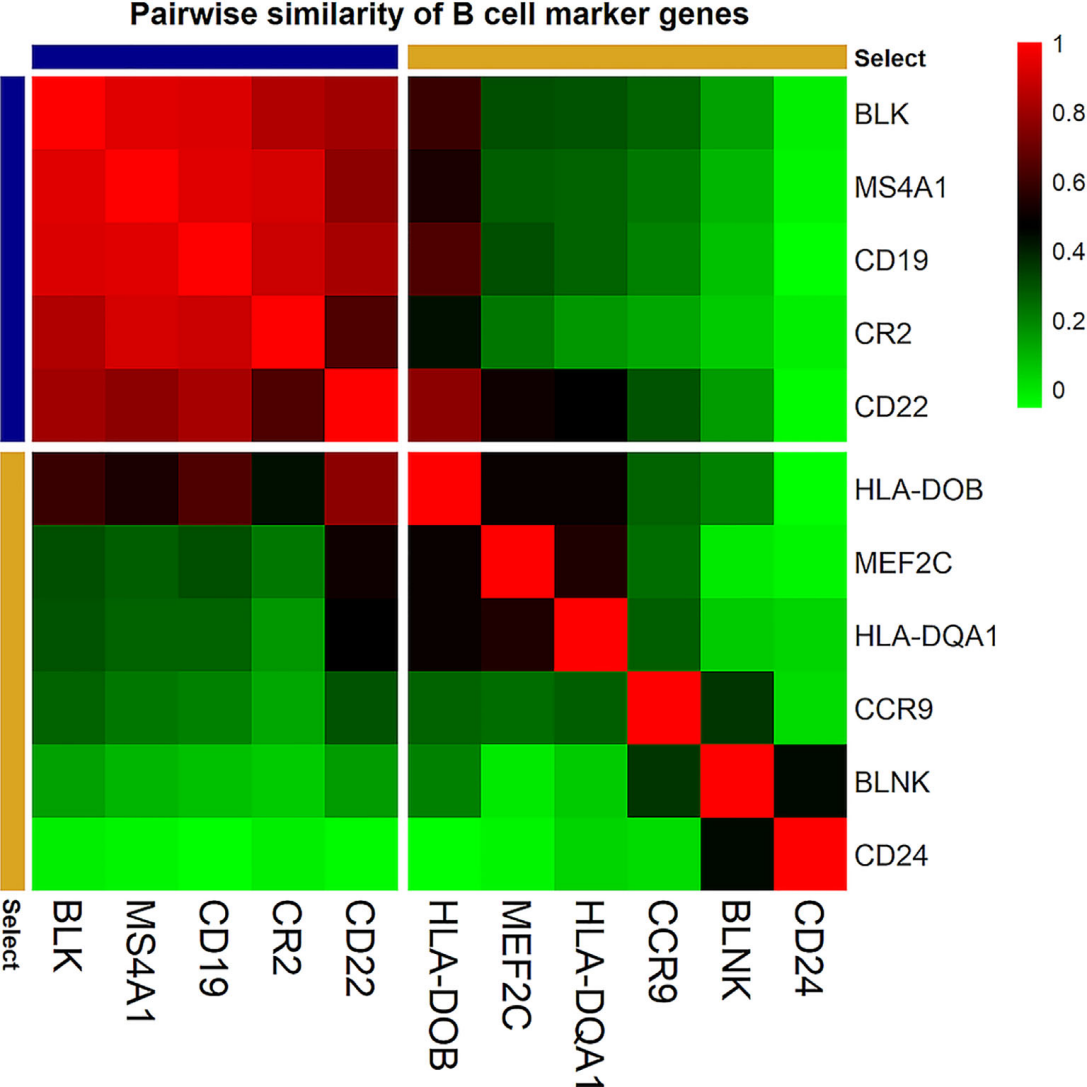
Correlation plot of the pairwise similarity of candidate marker genes tested to identify B cells. The pairwise similarity varies between the 11 selected genes. Five genes (blue) showed a high pairwise similarity (≥ 0.6). These genes were selected to identify B cell infiltration in PDAC tissue samples. Six genes (yellow) showed a low pairwise similarity (< 0.6). these genes were not used to identify B cell infiltration in PDAC tissue samples. The red color in the correlation plot presented the highest correlation score between the genes (R^2 ^= 1); the green color presented the lowest correlation score (R^2 ^= 0).

The PDAC-cMGs with a pairwise similarity ≥ 0.6 were selected and summarized in (**Table 1** **, Column 3**). For example, 11 genes were chosen as candidate genes to define B cells. Calculating the pairwise similarity of all the 11 genes showed that 5 genes had a high pairwise similarity (≥ 0.6), while the other 6 genes had a low pairwise similarity (< 0.6). Therefore, B cells were defined using the 5 genes with high pairwise similarity (**Figure 2**). Following the same method, a total of 55 genes were selected as marker genes to identify 22 immune cells in PDAC (**Supplementary File 1**).

**Figure 3:**
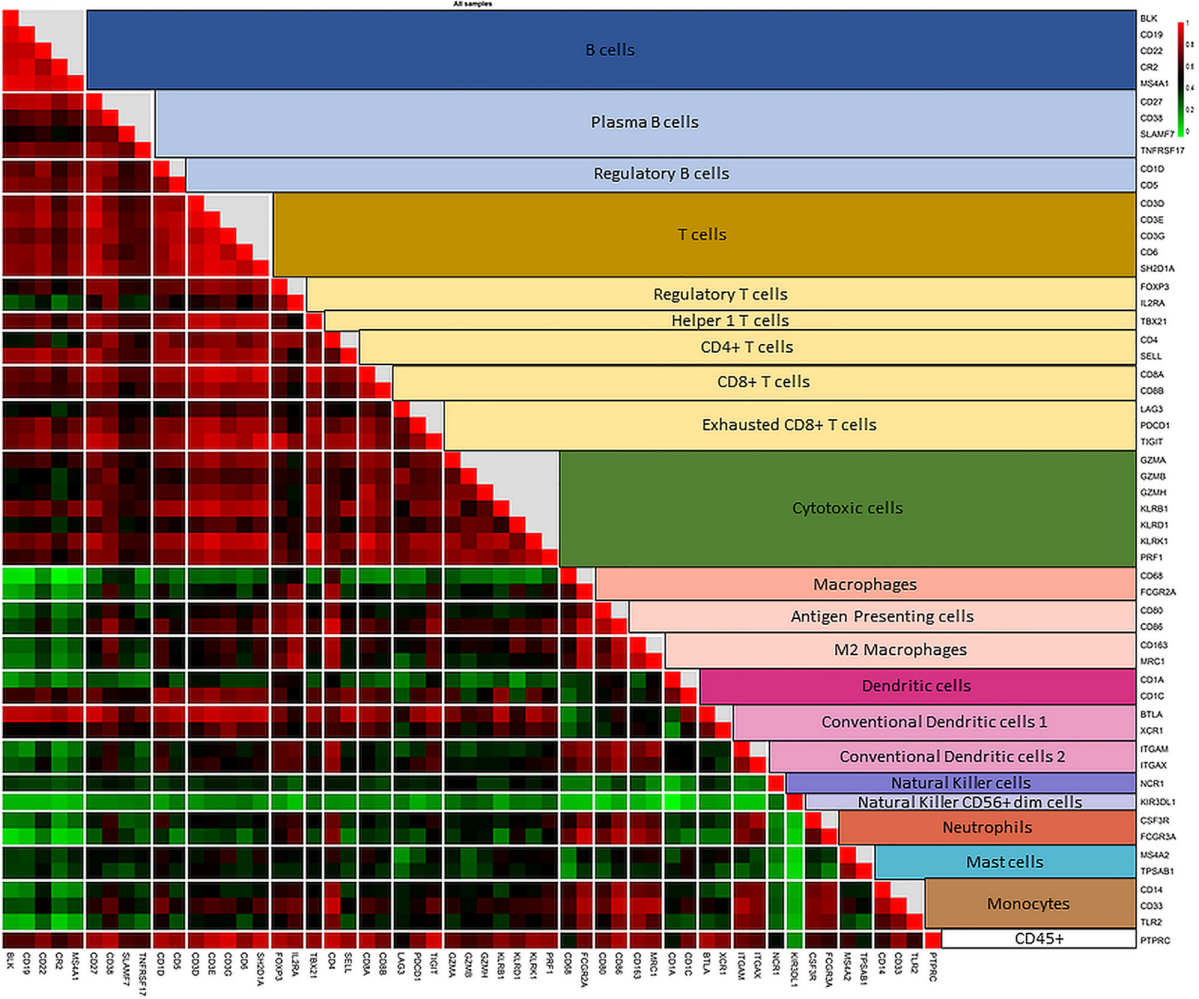
Correlation plot of the pairwise similarity of all 55 marker genes selected to identify the immune repertoire in PDAC tissue sample. Pairwise similarity plot shows a high correlation between marker genes that identify a specific immune family and the subtype of that family. The highest correlation is shown between the marker genes that identify a specific type of immune cell. In addition, a relatively high correlation is shown between the subtypes of immune cells of the same family (B cells and various subtypes of B cells; T cells and various subtypes of T cells). The correlation between T cells and cytotoxic cells is lower than the other subtypes of T cells because cytotoxic cells include both T and NK cells. The correlation plot also shows a high pairwise similarity and a high specificity of marker genes that identify macrophages and their subtypes in PDAC tissue samples. However, the various types of dendritic cells (DCs)are more difficult to identify. Genes used to identify DCs show a good correlation with T cells and macrophages, highlighting the need to use other marker genes (not measured by the PanCancer Immune profile panel) to increase the accuracy of identifying DCs in PDAC tissue samples.

Enriching the PDAC-MGICs list by additional genes from the literature enabled identifying 8 additional immune-cells that were not included in the default setting of nSolver^®^ Advanced Analysis module. These cells are plasma B cells, regulatory B cells (Bregs), 2 types of conventional dendritic cells (cDC), antigen-presenting cells (APCs), M2 macrophages, monocytes, and CD4+ T cells. In addition, the enrichment of the PDAC-MGICs increased the accuracy to identify Tregs cells, B cells, and macrophages, because more genes were used to identify these cells as compared to the default settings. To validate the specificity of the selected markers, pairwise similarities were calculated across all marker genes. The results are shown as a correlation plot for all the marker genes (**Figure 3**). The highest correlation was achieved between marker genes that were used to identify a specific immune cell. However, a relatively high correlation was also seen across other types of cells. For example, the 5 genes that identified B cells showed the highest correlation for B cells. But they also showed a lower but still good correlation in identifying T cells. This highlights the importance of trusting marker genes that have the highest pairwise similarity to identify a specific cell type. Marker genes that were used to identify cDC2 showed a relatively high correlation in Monocytes and M2 macrophages, highlighting that the definition of cDC2 is challenging using gene expressions of the PanCancer Immune profile panel in PDAC tissue samples, and can be improved by selecting additional marker genes.

### Validation of PDAC Marker Genes

To validate the accuracy of the PDAC-MGICs, the mean pairwise similarities between the corresponding marker genes were calculated in the TCGA PAAD dataset (**Table 2** **, Column 2**) and the Surgery Only dataset (**Table 2** **, Column 4**). These similarities were compared to those calculated between the cell types defined by the default gene markers in nSolver^®^ Advanced Analysis (**Table 2** **, Column 3 & 5**). Using the PDAC-MGICs resulted in an improved pairwise similarity (≥ 0.6) in both datasets for B cells, cytotoxic cells, DCs, neutrophils, and T cells. From the eight newly defined immune cells, five have a mean pairwise similarity ≥ 0.6 in both datasets. The exceptions were Bregs, and the 2 types of cDCs. Furthermore, the concordance per cell type of the PDAC-MGICs in the Surgery Only was calculated (**Table 2** **, Column 6**) and was compared to the default gene markers (**Table 2** **, Column 7**). The *p*-value for concordance improved for all PDAC-MGICs compared to the default markers in nSolver^®^ except for macrophages, CD8+ T cells, and exhausted CD8+ T cells.

**Table 2:** The pairwise similarities and concordance *p*-values of the PDAC-MGICs compared to the default marker genes of nSolver^®^ software, Advanced Analysis module.

	Column 2	Column 3	Column 4	Column 5	Column 6	Column 7	Column 8	Column 9	Column 10	Column 11
Cell type	PDAC-MGICs mean pairwise similarity in TCGA PAAD dataset	Default marker genes mean pairwise similarity in TCGA PAAD dataset	PDAC-MGICs mean pairwise similarity in GSE129492 Surgery Only	Default marker genes mean pairwise similarity in GSE129492 Surgery Only*	PDAC-MGICs concordance in GSE129492 Surgery Only	Default marker genes concordance in GSE129492 Surgery Only*	PDAC-MGICs mean pairwise similarity in GSE129492 Neoadjuvant	Default marker genes mean pairwise similarity in GSE129492 Neoadjuvant*	PDAC-MGICs concordance in GSE129492 Neoadjuvant	Default marker genes concordance in GSE129492 Neoadjuvant*
**B cells**	0.84	0.71	0.92	0.87	0.00	0.00	0.70	0.59	0.00	0.01
**Plasma B cells**	0.71		0.90		0.00		0.71		0.00	
**Regulatory B cells**	0.72		0.44		0.24		0.60		0.06	
**Cytotoxic cells**	0.7	0.64	0.59	0.54	0.00	0.01	0.43	0.46	0.01	0.01
**Dendritic cells**	0.7	0.48	0.80	0.19	0.04	0.43	0.37	0.08	0.21	0.58
**Conventional Dendritic cells 1**	0.68		0.53		0.18		0.71		0.02	
**Conventional Dendritic cells 2**	0.75		0.48		0.21		0.37		0.20	
**Macrophages**	0.55	0.64	0.49	0.55	0.23	0.07	0.68	0.59	0.03	0.01
**Antigen presenting cells**	0.8		0.85		0.02		0.58		0.08	
**M2 Macrophages**	0.84		0.67		0.09		0.76		0.01	
**Mast cells**	0.73	0.76	0.67	0.67	0.09	0.10	0.59	0.59	0.08	0.06
**Monocytes**	0.71		0.62		0.04		0.48		0.06	
**Natural Killer cells**		0.42		0.25		1.00		0.45		0.18
**Natural Killer CD56+ dim cells**		0.26		0.37		0.70		0.28		0.13
**Neutrophils**	0.67	0.52	0.68	0.46	0.09	0.22	0.24	0.43	0.35	0.16
**T cells**	0.88	0.87	0.82	0.82	0.00	0.00	0.51	0.51	0.01	0.01
**CD4+ T cells**	0.61		0.66		0.10		0.48		0.14	
**CD8+ T cells**	0.86	0.86	0.07	0.07	0.58	0.546	0.73	0.73	0.02	0.02
**Exhausted CD8+ T cells**	0.68	0.43	0.54	0.59	0.11	0.074	0.21	0.06	0.35	0.56
**Regulatory T cells**	0.73		0.81		0.04		0.70		0.04	

Asterisk (*) Only genes available in the PanCancer immune profiling panel (Platform GPL19965) are used to calculate the pairwise similarity. Underlined cell types are newly defined in comparison to the default of nSolver^®^ Advanced Analysis module of NanoString technology.

### Validation of PDAC-MGICs in PDAC Samples Subjected to Neoadjuvant Therapy

The usability of the PDAC-MGICs was checked by calculating the pairwise similarity (**Table 2** **, Column 8**) and concordance (**Table 2** **, Column 10**) in 18 samples of patients that received neoadjuvant therapy prior to surgery and compared to the default gene markers (**Table 2** **, Column 9, 11**). Similar results to Surgery Only group were achieved. An improvement of the pairwise similarity and concordance was shown for all PDAC-MGICs except for neutrophils that were not robustly identified in the default settings.

### Utilization of PDAC Marker Genes

**Figure 4:**
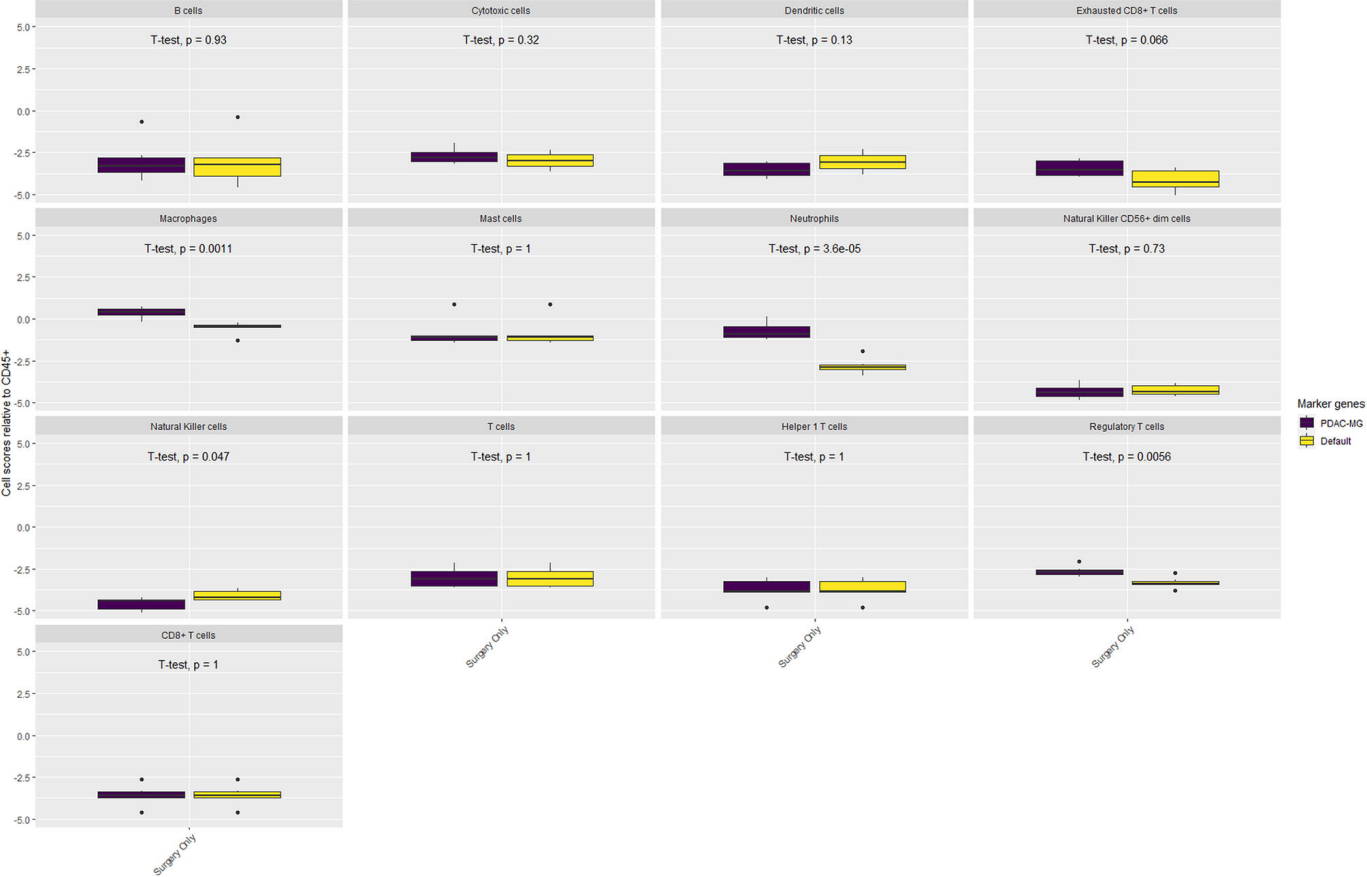
The impact of using PDAC-MGICs to identify immune cells in PDAC tissue samples. Comparing the relative immune scores using mRNA expression data of 6 tissue samples of patients who were subjected to surgery before receiving any treatment (Surgery Only). Immune cells were identified using the PDAC-MGICs set (purple) or the default marker genes in nSolver^®^ Advanced Analysis module of NanoString technology (yellow). All cell types were relative to the total infiltration of CD45+ expression. Identifying immune cells based on the PDAC-MGICs shows a significant variation (*p-value* < 0.05) in Macrophages, Neutrophils, Natural Killer cells, and Tregs cells.

**Figure 5:**
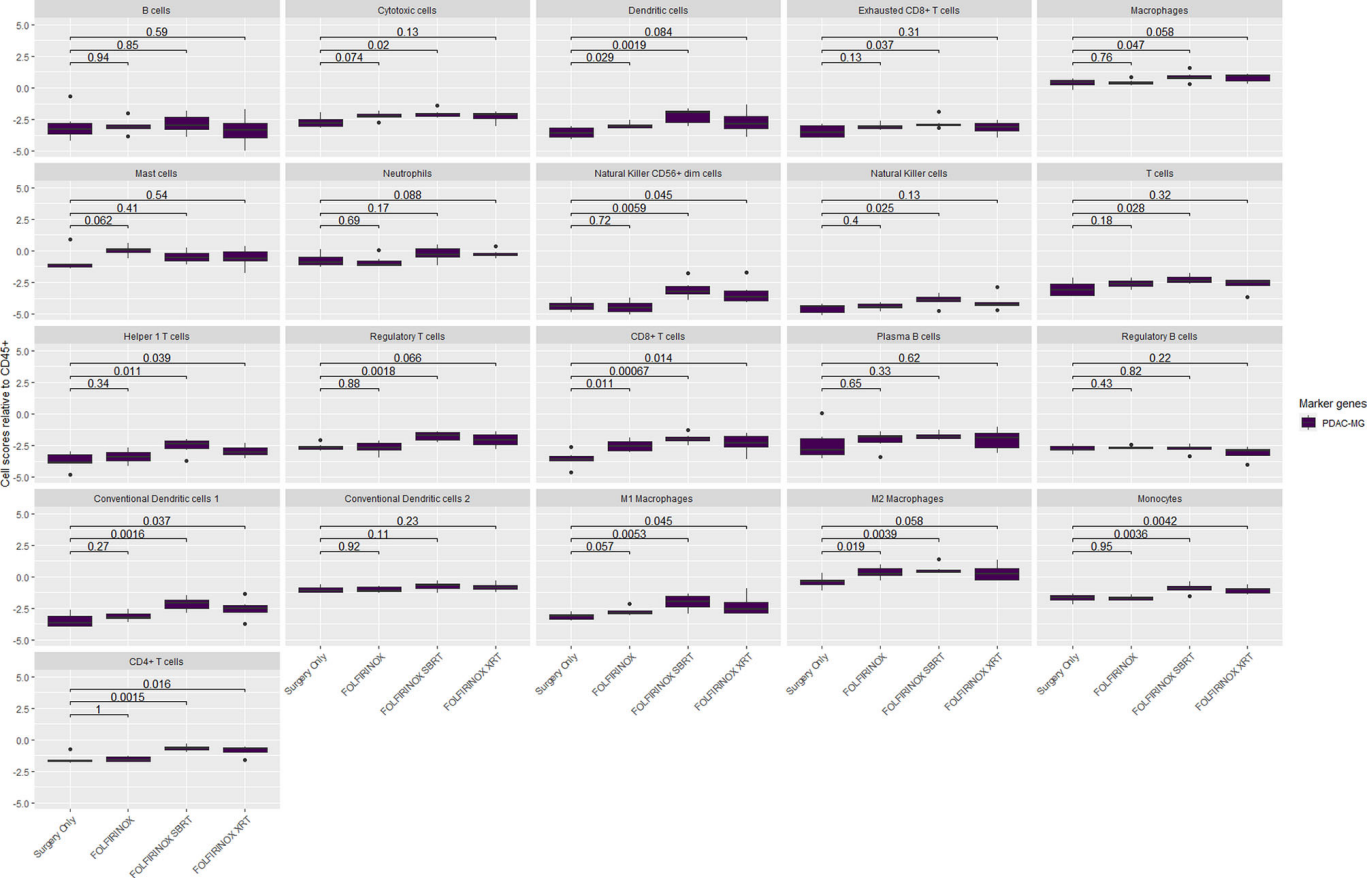
The relative immune abundance in PDAC tissue samples that received neoadjuvant therapy compared to treatment naïve samples using PDAC-MGICs. Comparing the relative immune scores using mRNA expression data of 18 PDAC tissue samples of patients who receive three types of neoadjuvant therapy compared to patients who were subjected to surgery before receiving any treatment (Surgery Only). Immune cells were identified using the PDAC-GMICs set and were presented relative to the total infiltration of CD45+ expression. The treatment effect of FOLFIRINOX + SBRT treated samples is most apparent. The *p*-values are the result of two-sided t-tests between Surgery Only and the other treatment groups individually. Surgery only (purple); neoadjuvant FOLFIRINOX (blue), FOLFIRINOX + SBRT (green), FOLFIRINOX + XRT (yellow).

The composition of the immune microenvironment for all samples published previously (41) was assessed by the nSolver^®^ Advanced Analysis module (NanoString). The relative abundance of the immune cell types is compared between PDAC-MGICs and the default marker genes in the Surgery Only samples (**Figure 4**). Defining immune cells based on the PDAC-MGICs showed a significant effect in the relative scores of macrophages, neutrophils, natural killer cells (NK) and Tregs compared to the default settings (**Figure 4**). The effect of neoadjuvant therapy on the relative scours of cells was tested and shown in (**Figure 5**). Defining immune cells using PDAC-GMICs revealed that FOLFIRINOX + SBRT had the biggest effect on immune cells compared to Surgery Only group. The results indicate an elevation of the cells as an effect of various types of neoadjuvant treatments, except for Bregs. The results are coherent with the previously reported results (41), but more immune sub-types were identified.

## Discussion

We have identified and validated specific marker genes to define immune cells in PDAC tissue samples (PDAC-MGICs). The PDAC-MGCI are more PDAC specific than the marker genes used to define immune cells across various types of tumor tissue samples (PanCancer marker genes). In addition, PDAC-MGICs enabled identifying eight additional immune cells (**Table 1**). To the best of our knowledge, our method is the only PDAC specific method that enables identifying 22 immune cells from 730 genes only. Moreover, it is the only method to describe the effect of (neo)adjuvant therapy in all 22 immune cells of PDAC tissue samples.

The method we provided is adapted from the previously published method based on the mathematical calculation of the pairwise similarities between the marker genes (16). Our method is based on using genes that are expressed above the background threshold. It differs in the number of genes used to identify immune cell types. We identified cells based on using at least two unique marker genes for each cell type. In addition, to increase the accuracy of cell definition, we chose a higher cut-off for the pairwise similarity (≥ 0.6) (42) compared to > 0.2 that was used in the previous publication. By increasing the threshold of the pairwise similarity, some important genes may not be used to identify an immune cell type. However, the accuracy of the identified immune cells will increase, which will be reflected on the time and money that will be spend on validating immune cells. The threshold can be adjusted to different levels in each experiment. We used ≥ 0.6 in order to identify immune cells with high level of accuracy that will minimize the amount of future validation. The set of genes used to identify immune cells has been reported to be expressed by a specific type of immune cells and showed a similar pattern of expression in PDAC database, which increases the cumulative evidence to be included as a marker gene in the PDAC-MGIC. The identification of immune cells infiltrating the tumor is very important to understand the underlying mechanisms of tumor immunogenicity (5, 6). While the previously described PanCancer marker genes (16) can give a comprehensive understanding of the relative immune cells’ abundance in various types of tumor tissue samples, it is highly important to check the pairwise similarity in a given database to ensure the accurate definition of immune cells. This importance becomes clear by checking DCs. The previously reported marker genes for DCs are shown to be insufficient for PDAC samples in contrast to pan-cancer samples (**Table 1**). Incorporating the PDAC-MGIC in nSolver^®^ Advanced Analysis software enabled discovering the effect of neoadjuvant therapy on the immune profiling of PDAC tissue samples. Our method showed the same effect of neoadjuvant therapy in PDAC samples as was reported before (41). However, it highlighted more clearly that the addition of a radiotherapy regimen to FOLFIRINOX induces more profound changes in gene expression than FOLFIRINOX alone. This was reflected in the relative scores of B cells, exhausted CD8+ T cells, macrophages, and neutrophils. The same types of cells had similar scores comparing Surgery Only group to FOLFIRINOX group, (**Supplementary Figures S1** **–** **S4**). Taken together, the results indicate that the addition of radiotherapy is necessary to stimulate immune cell infiltration in PDAC patients.

It should be noted that our method should be used to describe the relative scours of immune cells in two or more groups of samples, but it does not support estimating the absolute number of immune cells. Using a gene expression-based method to identify immune cells does not allow distinguishing between the number and the activity of cells. In addition, the definition of immune cells based on using one marker gene only like NK cells, NK CD56+ dim cells, and Helper 1 T cells, or cells that showed pairwise similarity < 0.6, remains not very accurate. However, in this study, we showed that the pairwise similarity is consistent between different databases (**Table 2**). Few exceptions were found, for example in CD8+ T cells, which highlights the huge effect of neoadjuvant therapy on the expression of genes that identify CD8+ T cells. CD8+ T cells were identified by using very specific and accurate genes: CD8A and CD8B genes. Therefore, the results reflect the effect of neoadjuvant therapy on the relative scours of CD8 cells. A recent publication described the immune landscape by estimating 22 different immune cells in PDAC samples using CIBERSORTx (43). The immune estimation was correlated to the molecular subtypes and the survival of the patients. Interestingly, the number of estimated immune cells was the same as we identified in our method. However, the immune subtypes do not completely overlap (**Supplementary Table S1**). In the study of Liu et al. (43), immune cells were computed by using LM22 gene signature containing 547 genes as reference. Opposite to our method, genes are not mutually exclusive. Although, an assumption is made by using mutually exclusive genes, our method can be used to estimate the relative abundance of 22 immune cells using 55 genes only. Furthermore, all marker genes described in our method are specifically measured in PDAC tissue samples, contrary to the LM22 gene signature reference. In addition, our method can be applied using gene expression data generated from samples that were preserved differently like FF and FFPE tissue samples or blood samples. Identifying PDAC specific immune cells using the PDAC-MGICs enables revealing the effect of any type of therapy in various clinical settings and clinical trials. Moreover, applying the method on data generated from blood samples supports monitoring the progression of patients, and maybe informative to direct therapeutic decisions.

Our method is easily tailored and applicable to identify specific immune cells in any type of tissue samples. Nevertheless, we highlight the importance of selecting and testing the marker genes critically for each tissue type. It has been shown that marker genes can be specified for each type of tissue samples (17). The candidate marker gene list can be checked and narrowed down to a more specific marker gene list by calculating the pairwise similarity between all pairs of genes to ensure the accurate identification of immune cells in any type of tissue samples. Once the marker genes for each immune cell have been identified and checked, the expression of the genes for each immune cell type can be compared between the groups of interest. This method can be applied using any RNA databases (sequencing or gene expressions). The use of single-cell sequencing has shown that cells of the same type can have different gene expression present (44). Furthermore, the assumption that the gene markers are exclusively expressed by one specific cell type is in many cases hard to prove. Therefore, we believe that the described method can accurately estimate the relative score of immune cells based on their marker genes definition.

## Conclusion

We provided a method to identify specific immune cells in PDAC tissue samples based on using mRNA expression of marker genes (PDAC-MGICs). In addition, we validated and utilized the PDAC-MGICs to delineate the effect of various (neo)adjuvant treatments on the immune landscape in PDAC tissue samples. The PDAC-MGICs set reflects the immune microenvironment of PDAC tumor tissue sample, however, it can be easily tailored and applicable to identify specific immune cells in any type of tissue samples.

## Data Availability Statement

The original contributions presented in the study are included in the article/**Supplementary Material**. Further inquiries can be directed to the corresponding author.

## Author Contributions

WD: literature search, figures, study design, data collection and analysis, data interpretation, and writing. DL: data collection, writing, and reviewing. YL: data analysis, writing, and reviewing. CV: supervising, writing, and reviewing. AS: literature search, study design, writing, and reviewing. DM: literature search, study design, data collection, data analysis and interpretation, writing, and reviewing. All authors contributed to the article and approved the submitted version.

## Funding

This project was made possible with the financial support of Support Casper Foundation (www.supportcasper.nl).

## Conflict of Interest

The authors declare that the research was conducted in the absence of any commercial or financial relationships that could be construed as a potential conflict of interest.

## Supplementary Material

Supplementary Table 1 The 22 immune cells estimated in PDAC samples using PDAC-MGICs and LM22 gene signature (CIBERSORTx).

Supplementary File 1 Detailed information about all marker genes that were chosen as candidate genes (PDAC-cMG), and the selected marker genes to identify immune cells in PDAC (PDAC-MGICs). The file contains all the pairwise similarity values between genes used to identify the immune cells in PDAC tissue samples. Genes highlighted with green were selected to identify immune cells; genes highlighted with red were not selected. The pairwise similarity values ≥ 0.6 were highlighted with gray color.

Supplementary Figure 1 The relative scores of B cells in 4 types of PDAC tissue samples of patients who were subjected to (neo)adjuvant therapy. The relative scours of B cells were calculated using the PDAC-MGIC (purple), or the default genes in nSolver^®^ software, the Advanced Analysis module (yellow).

Supplementary Figure 2 The relative scores of exhausted T cells in 4 types of PDAC tissue samples of patients who were subjected to (neo)adjuvant therapy. The relative scours of exhausted T cells were calculated using the PDAC-MGIC (purple), or the default genes in nSolver^®^ software, the Advanced Analysis module (yellow).

Supplementary Figure 3 The relative scores of macrophages in 4 types of PDAC tissue samples of patients who were subjected to (neo)adjuvant therapy. The relative scours of macrophages were calculated using the PDAC-MGIC (purple), or the default genes in nSolver^®^ software, the Advanced Analysis module (yellow).

Supplementary Figure 4 The relative scores of neutrophils in 4 types of PDAC tissue samples of patients who were subjected to (neo)adjuvant therapy. The relative scours of neutrophils were calculated using the PDAC-MGIC (purple), or the default genes in nSolver^®^ software, the Advanced Analysis module (yellow).
