# Supplementary figures and images for: Identification, Validation, and Utilization of Immune Cells in Pancreatic Ductal Adenocarcinoma Based on Marker Genes
Source: Front Immunol. 2021 Apr 27;12:649061. doi: 10.3389/fimmu.2021.649061 (PMC8110918; doi:10.3389/fimmu.2021.649061)

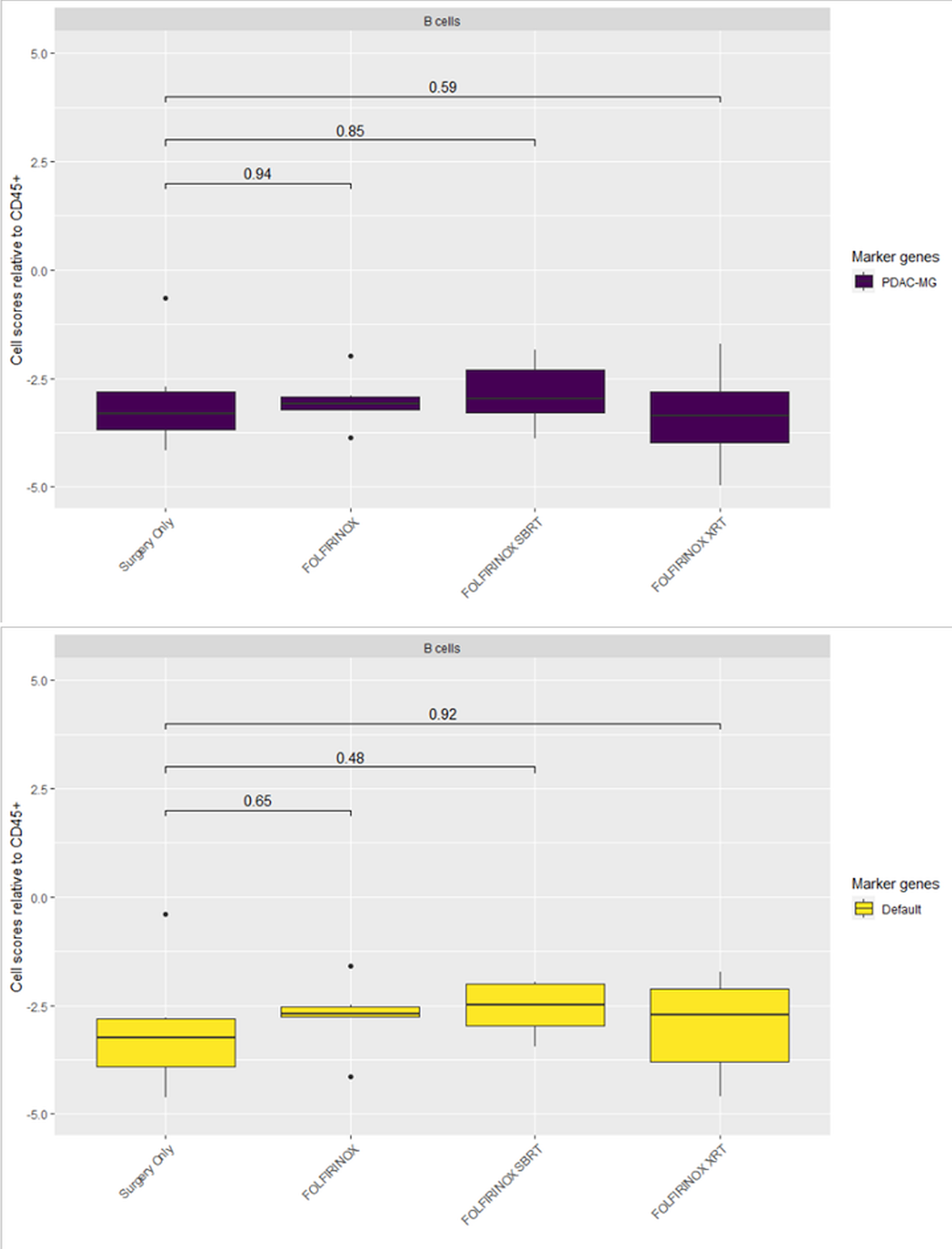

Supplement: Supplementary Figure 1 — The relative scores of B cells in 4 types of PDAC tissue samples of patients who were subjected to (neo)adjuvant therapy. The relative scours of B cells were calculated using the PDAC-MGIC (purple), or the default genes in nSolver® software, the Advanced Analysis module (yellow). [file Image_1.tif]

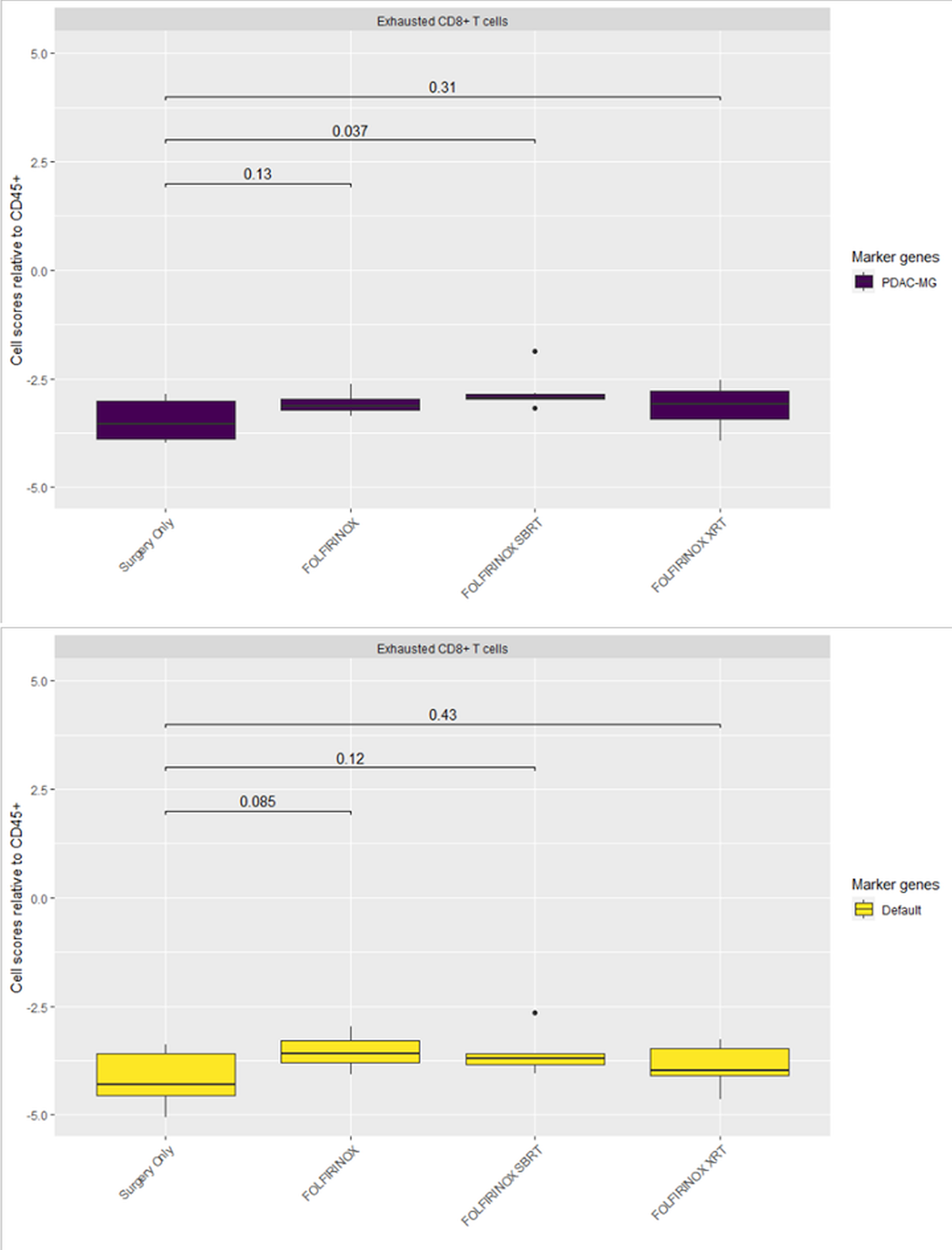

Supplement: Supplementary Figure 2 — The relative scores of exhausted T cells in 4 types of PDAC tissue samples of patients who were subjected to (neo)adjuvant therapy. The relative scours of exhausted T cells were calculated using the PDAC-MGIC (purple), or the default genes in nSolver® software, the Advanced Analysis module (yellow). [file Image_2.tif]

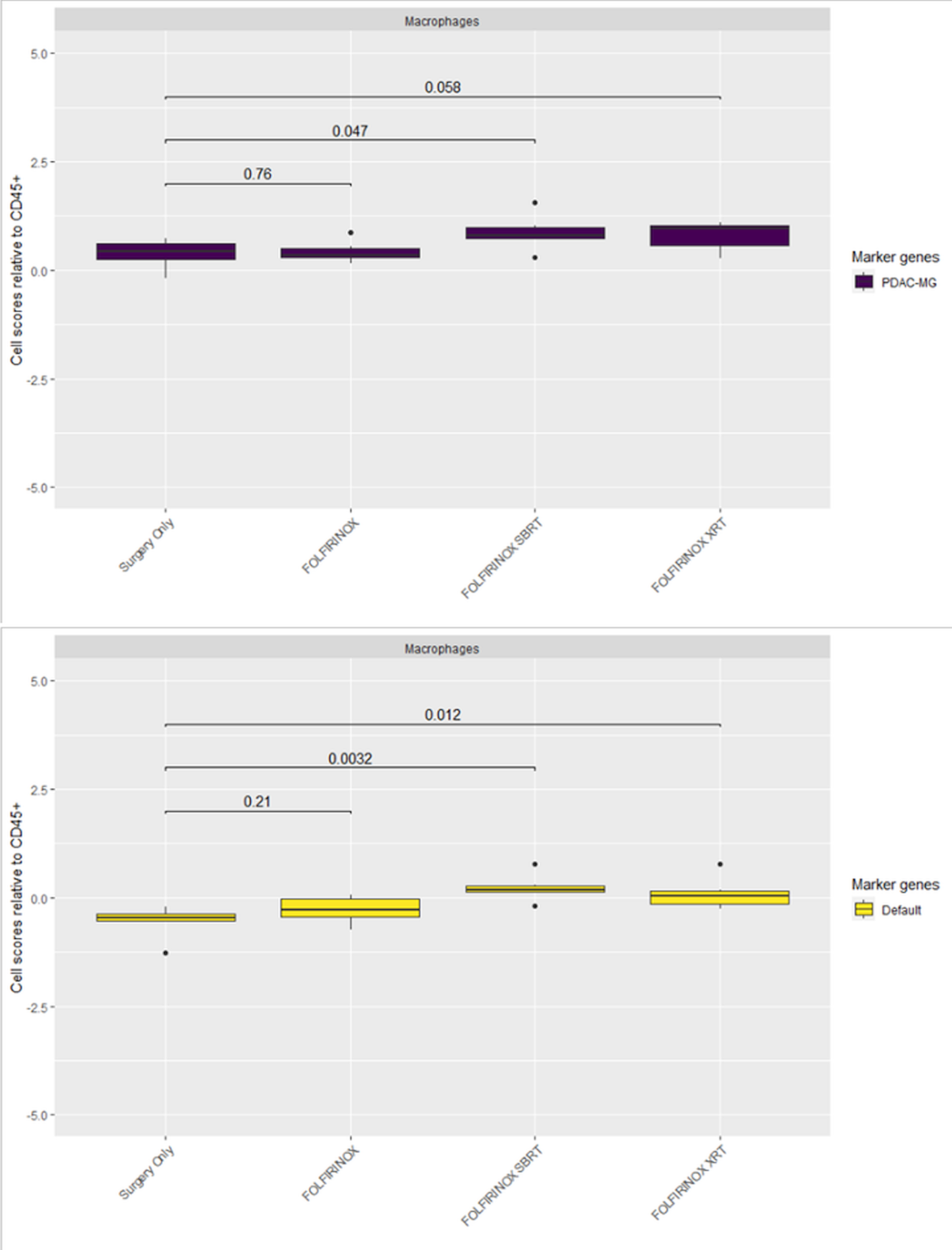

Supplement: Supplementary Figure 3 — The relative scores of macrophages in 4 types of PDAC tissue samples of patients who were subjected to (neo)adjuvant therapy. The relative scours of macrophages were calculated using the PDAC-MGIC (purple), or the default genes in nSolver® software, the Advanced Analysis module (yellow). [file Image_3.tif]

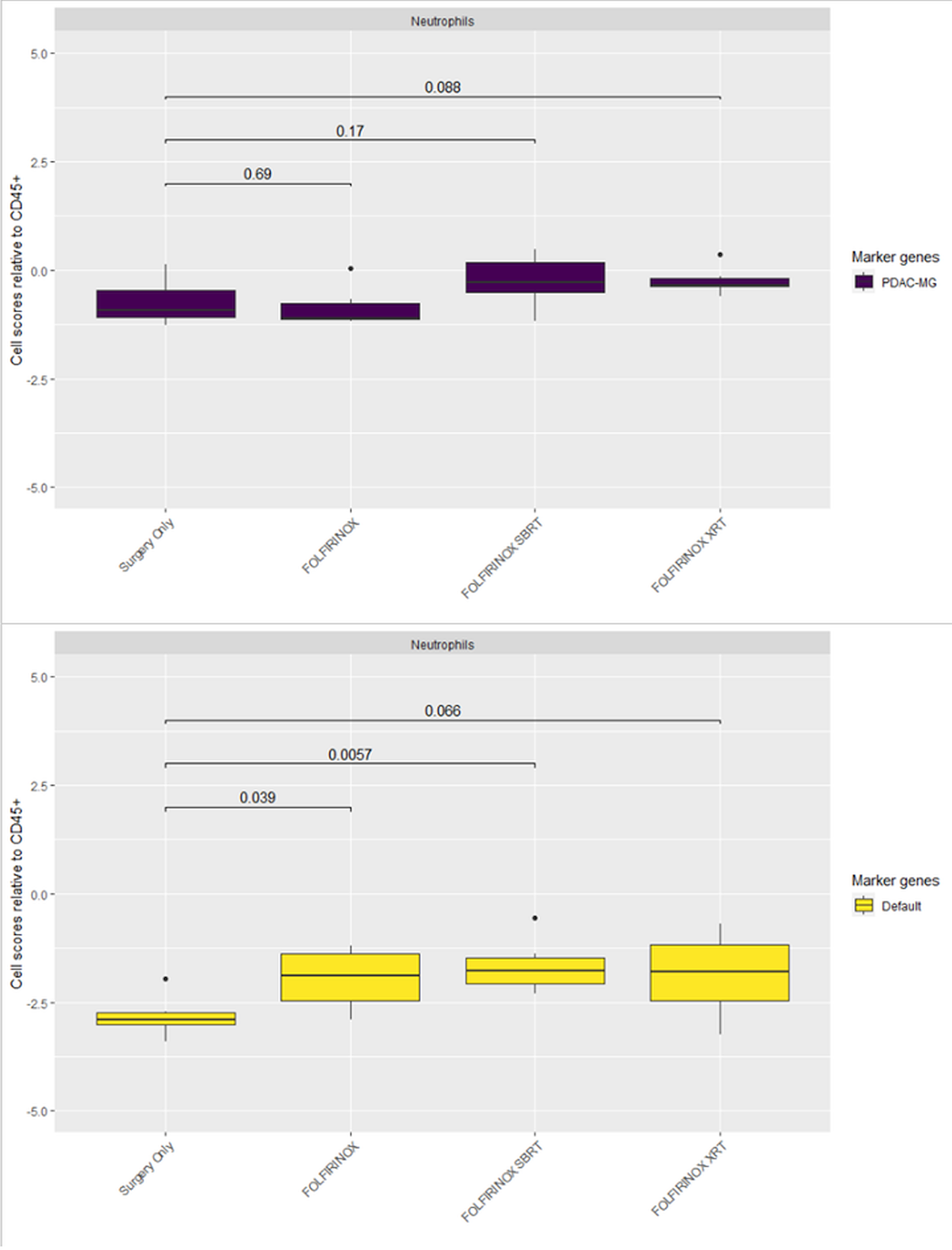

Supplement: Supplementary Figure 4 — The relative scores of neutrophils in 4 types of PDAC tissue samples of patients who were subjected to (neo)adjuvant therapy. The relative scours of neutrophils were calculated using the PDAC-MGIC (purple), or the default genes in nSolver® software, the Advanced Analysis module (yellow). [file Image_4.tif]
